# The Influence of Social Desirability on Sexual Behavior Surveys: A Review

**DOI:** 10.1007/s10508-021-02197-0

**Published:** 2022-02-10

**Authors:** Bruce M. King

**Affiliations:** Department of Psychology, 418 Brackett Hall, Clemson University, Clemson, SC 29634 USA

**Keywords:** Sexual behavior, Sexual behavior surveys, Response bias, Social desirability

## Abstract

Research in fields for which self-reported behaviors can be compared with factual data reveals that misreporting is pervasive and often extreme. The degree of misreporting is correlated with the level of social desirability, i.e., the need to respond in a culturally appropriate manner. People who are influenced by social desirability tend to over-report culturally desired behaviors and under-report undesired behaviors. This paper reviews socially desirable responding in sexual behavior research. Given the very private nature of the sexual activity, sex researchers generally lack a gold standard by which to compare self-reported sexual behaviors and have relied on the anonymity of participants as the methodology to assure honest answers on sexual behavior surveys. However, indirect evidence indicates that under-reporting (e.g., of a number of sexual partners, receptive anal intercourse, condom use) is common. Among the general population, several studies have now reported that even with anonymous responding, there are significant correlations between a variety of self-reported sexual behaviors (e.g., use of condoms, sexual fantasies, exposure to pornography, penis size) and social desirability, with evidence that extreme under- or over-reporting is as common as is found in other fields. When asking highly sensitive questions, sex researchers should always include a measure of social desirability and take that into account when analyzing their results.

## Introduction

From the time of the first survey by Kinsey et al. (1948) to the present, our knowledge of the incidence and prevalence of sexual behaviors has relied heavily on self-reports. Public policy with regard to reproductive and sexual health is based, in large part, on people’s answers on surveys of sexual behavior. Several weaknesses are recognized, including but not limited to the use of convenience samples and volunteer bias (Bogaert, 1996; Strassberg & Lowe, 1995), the type of assessment tool, terminology and question structure, and participation rate (Catania et al., 1990a, 1990b; Fenton et al., 2001). Self-reports can be flawed by forgetting and false memories (Archer et al., 2015; Catania et al., 1990a, 1990b). Of particular concern is response bias, a tendency to answer questions based on something other than the content (Paulhus, 1991). One of the most frequent types of response bias is socially desirable responding, i.e., “the need of [individuals] to obtain approval by responding in a culturally appropriate manner” (Crowne & Marlowe, 1960, p. 353). Put simply, if a respondent is influenced by social desirability, he or she may over-report desirable behaviors and under-report undesirable behaviors.

This paper reviews social desirability responding to self-reported sexual behaviors. The research literature from 1975 to the present was surveyed primarily with the use of Medline and Social Sciences with Full Text using “social desirability” and “response bias” as key terms. In addition, each issue of *Archives of Sexual Behavior* and *Journal of Sex Research* were examined for pertinent articles.

## Social Desirability Responding in Other Fields of Research

Given the private nature of the sexual activity, sex researchers rarely are able to use factual information to which self-reported behaviors can be compared (Catania et al., 1990a, 1990b). We will never know for sure how many sexual partners an individual has had or the frequency with which he or she engages in sexual behaviors. Thus, it is important that sex researchers know that social desirability bias has been well-established in other areas of research that similarly rely on self-reported behaviors.

Consider, for example, the health field. Here, researchers are able to verify self-reports with actual intake (the gold standard). Under-reporting of energy intake by 30% or more is common (Archer et al., 2013; Lissner et al., 2007; Subar et al., 2003), with at least 10–14% of people under-reporting to such an extent that they are referred to as “extreme under-reporters” (Ferrari et al., 2002). The degree of under-reporting increases with each incremental increase in BMI (Braam et al., 1998). This cannot be attributed entirely to false recall as individuals with clinical obesity also under-report when taking an inventory of high-calorie foods in their homes (King et al., 2016). Statistically significant correlations are found between the degree of actual under-reporting (self-reported values minus actual values) and measures of social desirability (Hebert et al., 2001, 2002; Scagliusi et al., 2003, 2009; Taren et al., 1999; Tooze et al., 2004). It has long been known that many people under-report their body weight and over-report their height as well (e.g., Brener et al., 2003; Connor Gorber et al., 2007; Merrill & Richardson, 2009; Nyholm et al., 2007; Palta et al., 1982), and that there is “robust evidence of social desirability bias” (Burke & Carman, 2017, p. 198). In a review of research of energy intake, one group of researchers concluded that data acquired by self-reports “are fundamentally and fatally flawed” (Archer et al., 2015, p. 911), and another review called self-reported data “implausible” (Ioannidis, 2013).

These conclusions are not limited to nutrition and obesity research. High levels of social desirability have also been found to be associated with under-reporting of alcohol and drugs (Davis et al., 2010; Latkin et al., 2017), heroin craving by former users (Marissen et al., 2006), and reckless driving by young adults (Bradley & Wildman, 2002).

Thirty-seven years ago, in a review of self-reported data in many fields (including anthropology, psychology, child care, and criminal justice), Bernard et al. (1984) concluded that the “results of all these studies lead to one overwhelming conclusion: on average, about half of what informants report is probably incorrect in some way” (p. 503) and that “informant inaccuracy remains a well-kept open secret” (p. 504).

## Social Desirability Responding in Sex Research

Although sex researchers usually lack the ability to factually check self-reported behaviors, there is ample indirect evidence that social desirability influences answers to questions on sex surveys. Early studies found high correlations between the probability of true responses to (a variety of) behavioral items and the judged social desirability of the item (Cruse, 1965; Edwards, 1970). Galbraith et al. (1974) extended this to sexual behaviors and found a correlation of 0.70, indicating a strong influence of social desirability on answers to questions about sexual behaviors.

Several studies of men who have sex with men have found evidence that social desirability bias affected answers to questions about HIV serostatus, a number of sexual partners, receptive anal intercourse, and needle injection (Gibson et al., 1999; Latkin & Vlahov, 1998; Latkin et al., 1993; Rao et al., 2017). The result is the under-estimation of those sexual behaviors. What about surveys given to the more general population?

In a 1966 study using the personal interview technique, researchers found that 7% of participants initially admitted to same-sex sexual experiences, but many others later changed their answers (resulting in 22%) when they were told that they would be given a polygraph test to detect false answers (Clark & Tiffit, 1966). Same-sex sexual relations were a highly stigmatized behavior in 1966 (see Editorial, 1966).

In another early study, researchers asked women in several repeated personal interviews if they had ever engaged in anal intercourse (Bolling, 1976; Bolling & Voeller, 1987). Very few admitted to doing so in the first interview, but after repeated interviews with the same researcher (and the “development of strong trust”) nearly three-fourths admitted to having tried it at least once.

With the use of daily sexual activity diaries as the gold standard, researchers have confirmed that many people over- or under-report sexual behaviors (Graham et al., 2003; McAuliffe et al., 2007). Many men over-report the use of condoms (Davoli et al., 1992; Zenilman et al., 1995). One study found a correlation of only 0.51 between men’s self-reported condom use and their partners’ reports for the previous 30 days (Ellish et al., 1996). These misreportings could be due to false recall and other factors, not just social desirability bias. However, one study found that high social desirability scores were associated with participants’ self-reports of always using condoms (Rao et al., 2017).

Social desirability has also been found to influence answers about extramarital affairs (Zapien, 2017) and self-reported exposure to pornography (Rasmussen et al., 2018). An early study found that heterosexual male gender patients who sought sex reassignment surgery exaggerated their admirable personal qualities during interviews with the staff (Blanchard et al., 1985).

Even some people’s definition of “sex” or “had sex” is influenced by social desirability. In a study of college students who admitted to having engaged in oral-genital contact but not intercourse, some admitted to having had sex while others denied it. The latter group scored significantly higher in social desirability (Den Haese & King, 2022).

Factor analysis has revealed that socially desirable responding has two components: self-deception (i.e., the respondent has an overly positive impression of himself or herself and believes his or her self-reports) and impression management (i.e., the respondent consciously misreports to deceive others) (Paulhus, 1984). The former is resistant to change, whereas impression management is influenced by the level of demand to present oneself positively.

Meston et al. (1998) found significant negative correlations between impression management social desirability scores and several sexuality self-reports (unrestricted sexual attitudes and fantasies for men; unrestricted sexual fantasies, sexual drive and experience, and virginity status for women), even after personality and conservatism factors were partialed out. The correlations were of the same magnitude as has been reported for energy intake studies.

As was found for nutritional self-reports, there is evidence of extreme misreporting on sexual behavior surveys. Men’s self-reports of erect penis size offer a good example. In several studies, the mean length for self-reported erect penis length has been 6.0–6.4 inches. The combined mean length in 10 studies in which researchers took measurements (of pharmacologically induced full erections) was 5.36 inches, and 5.11 inches in 21 studies of fully stretched penises (King, 2021). In a recent study, 10% of sexually experienced men self-reported erect penis lengths of 8 inches or more (and 30.8% reported 7 inches or more). The correlation with Marlowe-Crowne social desirability scores was 0.26 (King et al., 2019). If actual error scores (self-reported length minus factual data) could have been collected as in nutrition research (e.g., Archer et al., 2013), the magnitude of the correlations would likely have been even greater.

It is important to recognize that the impression management component of socially desirable responding is different from the intentional mischievous responding that has recently been reported with surveys of sexual minority youth (Cimpian & Timmer, 2020; Fish & Russell, 2018). In this example, individuals have misidentified themselves as a sexual minority with the intention of distorting results.

## Conclusions

In a recent review, Schmitt (2017) concluded, “In the end, ample research suggests responses to sexuality surveys are….mostly truthful” (concluding paragraph). This author disagrees. For example, the CDC’s Youth Risk Behavior Survey (YRBS) is a national school-based survey of a large variety of self-reported risky behaviors among U.S. adolescents. Many researchers, including this author, have cited the results from the sexual behaviors portion of the survey. The 2015 survey has been cited over 1420 times (Kann et al., 2016) and the 2017 survey has been cited over 1,400 times (Kann et al., 2018). However, in a study of the validity of their findings, the CDC found that students over-reported their height by an average of 2.7 inches. The misreporting was not random. Only 4% of the participants under-reported their height, with 39.5% over-reporting by 3 inches or more (Brener et al., 2003). Mischievous responding was evident as well as one high school student over-reported height by 16.7 inches. With many of the same students under-reporting their body weight, 12.7% under-reported their body mass index by 5 kg/m^2^ or more.

There is no rational reason to believe that answers on the sexual behaviors portion of the YRBS, or any other survey of self-reported sexual behaviors, are any more truthful than the YRBS’ self-reports of height. In one of the few studies in which self-reported sexual behavior was compared to the gold standard of factual information, adolescents were asked if they had experienced a sexually transmitted infection in the previous 6 months to 1 year (Clark et al., 1997). Fifty-one percent denied having had an STI, but hospital records confirmed that they had. Another 9% admitted to having had one STI during that time period, but medical records revealed multiple STIs. The results of many studies now indicate that social desirability responding in studies of self-reported sexual behaviors is as pervasive and often as extreme as is found in other research areas.

## Recommendations

There are procedural and analytical steps that are intended to minimize the effects of response bias. Several studies have found that compared to face-to-face interviews and/or questionnaires on which respondents are required to give their name, results are more accurate when respondents answer questions anonymously under self-administered conditions (e.g., Durant & Carey, 2000; Robertson et al., 2018) and that self-administered tests have good test–retest reliability (Durant & Carey, 2002). However, carefully controlled studies reveal that anonymity alone has only a minimal effect on reducing social desirability responding (Dalal & Hakel, 2016). Extreme misreporting occurs even with anonymous, self-reported, paper-and-pencil questionnaires (King et al., 2019).

Researchers no longer need to rely on the paper-and-pencil techniques for self-reports of sexual behavior. Technological advances now allow for self-reports of sexual behavior via the Internet and computers (McCallum & Peterson, 2012). For example, the British National Survey of Sexual Attitudes and Lifestyles uses this methodology (Erens et al., 2014). Some studies initially reported that unproctored computer-assisted self-administered interviews, by providing greater privacy and anonymity, decreased social desirability bias (Ghanem et al., 2005; Kissinger et al., 1999). However, recent meta-analyses of web-based assessments indicate that social desirability responding was no less with computerized assessments than for paper-and-pencil surveys (Gnambs & Kaspar, 2017). In summary, although one can point to individual studies that claim web-based assessment decreases social desirability responding, the whole of these studies finds little to no effect.

Another technique to possibly reduce social desirability bias is indirect questioning, whereby people answer questions from another person’s or group’s perspective (Fisher, 1993). Dalal and Hakel (2016, p. 483) gave the following example: If a researcher is interested in the use of illegal drugs or alcohol on the job, the usual direct approach would be to ask the participant, “To what extent have you used an illegal drug or consumed alcohol on the job?” The indirect approach would phrase the question as “To what extent has the average worker in your workplace used an illegal drug or consumed alcohol on the job?” A recent meta-analysis of 143 studies (of ethical decision-making – all fields) showed indirect questioning to be more effective than the direct measurement approach in reducing social desirability bias (Yang et al., 2017). However, it is doubtful whether the indirect questioning technique can be used to answer many of the questions that may be of interest to sex researchers (e.g., frequency of masturbation, vaginal intercourse, anal intercourse, and a number of sexual partners).

A recent study suggested that social desirability bias in qualitative research can be minimized by training interviewers to identify word choice patterns and the nature of responses (Bergen & Labonte, 2020). This, of course, is very subjective.

Thus, regardless of the technique used, it is advised that when sex researchers ask participants sexually sensitive questions, a measure of social desirability should also be included. The most widely used measure has been the Marlowe-Crowne scale (Crowne & Marlowe, 1960), cited over 13,200 times. It is a 33-item True–False scale that is appropriate for all fields of research that rely on self-reported data. Here are three example statements: “I am always courteous, even to people who are disagreeable”; “No matter who I’m talking to, I’m always a good listener”; and “I’m always willing to admit it when I make a mistake.” If brevity is required, a 13-item short form is available that has been shown to have both high internal consistency and good test–retest reliability (Reynolds, 1982). Paulhus (2002) offered a 20-item measure for impression management that has been used for both interpersonal and intrapersonal sexual behaviors (Meston et al., 1998).

Paulhus (1991) suggested 30 years ago that raw scores could be adjusted by logistic regression. Gibson et al. (1999) were possibly the first to use this technique to calculate social desirability bias-free measures of self-reported sexual behaviors. In brief, their approach was “to measure socially desirable response tendency [using the Marlowe-Crowne scale] alongside a measure of interest and then adjust raw scores on that measure by an amount commensurate with the degree of socially desirable responding” (p. 97). Other studies have adjusted self-reported data in a similar manner (e.g., Rao et al., 2017), but it is not common. For example, similar to the YRSB, neither the National Survey of Sexual Health and Behavior (NSSHB) nor the National Survey of Family Growth (NSFG) included a measure of social desirability responding. This author recommends that it should now become standard practice, especially for large national surveys that are used to formulate public policy.

When presenting their data, researchers should consider including the median as a measure of central tendency. Unlike the mean, the median is unaffected by extreme scores (and thus, extreme under-reporting or over-reporting), as well as mischievous responding. For example, several sex surveys have found that the mean lifetime number of sexual partners reported by men greatly exceeds the number reported by women. This gender difference is not observed using a “bogus pipeline” technique in which respondents are connected to a fake polygraph that supposedly detects untruthful responses (Alexander & Fisher, 2003). The difference also disappears when one compares the medians (Conley et al., 2011; Pedersen et al., 2002), suggesting that the mean difference was due to (sometimes extreme) over-reporting by some men and/or under-reporting by some women.

The use of scales to measure overall social desirability bias in studies of self-reported behaviors is limited if subgroups within a sample have different ideas of what is socially desirable. For example, in order to adjust data to account for social desirability bias, it is important that we understand any differences among demographic groups. With regard to under-reporting of energy intake, it has been factually determined that it is more common among women than men, and among older persons than among younger people (see Tooze et al., 2004, for references). Meston et al. (1998) found some differences between college men and women in the types of self-reported behaviors that were correlated with social desirability. However, not enough large-scale research has been conducted with social desirability bias and self-reported sexual behaviors to know if there are consistent differences between major demographic groups.

Behavioral scientists have expressed concern about the possible influence of social desirability bias for nearly 90 years (Bernreuter, 1933). Reliable and valid measures to identify it have existed for over 60 years (e.g., Crowne & Marlowe, 1960). When asking individuals about their private sexual behaviors, attitudes, and desires, sex researchers should minimize social desirability responding (beyond anonymous responding) and attempt to ascertain the magnitude of any social desirability bias.
